# LC-MS/MS Quantification of Tramadol and Gabapentin Utilizing Solid Phase Extraction

**DOI:** 10.1155/2018/1605950

**Published:** 2018-10-28

**Authors:** Pappula Nagaraju, Balaji Kodali, Peda Varma Datla, Surya Prakasarao Kovvasu

**Affiliations:** ^1^Department of Pharmaceutical Analysis, Hindu College of Pharmacy, Guntur 522002, Andhra Pradesh, India; ^2^College of Pharmaceutical Sciences, Acharya Nagarjuna University, Nagarjuna Nagar 522510, Guntur, Andhra Pradesh, India; ^3^Clinical Pharmacology and Bio Sciences Division, RA Chem Pharma, Hyderabad, India; ^4^College of Pharmacy, Western University of Health Sciences, Pomona, CA 91766, USA

## Abstract

An accurate, highly sensitive, and precise method for quantitative analysis of tramadol (TMD) and gabapentin (GBP) by high performance liquid chromatography and tandem mass spectrometry in human plasma was proposed and validated successfully using venlafaxine and pregabalin as internal standards (ISTDs), respectively. An aliquot of 200 *μ*L of plasma was mixed with internal standard dilution and extraction was performed by using solid phase extraction (SPE) technique. Peak resolution was achieved on Phenomenex PFP column (50×4.6 mm, 2.6 *μ*m). The total analytical run time was 3.8 min. Both analytes were monitored using multiple reaction monitoring (MRM) scan and the mass spectrometer was operated in positive polarity mode. The method was validated for specificity, sensitivity, precision, accuracy, and other analytical parameters. The results found were satisfactory over the linear calibration range of 1-500 ng/mL and 10-6000 ng/mL for TMD and GBP, respectively. The developed method can be ready to use by scientific community for quantification of analytes in plasma samples from various clinical studies of different dose strengths.

## 1. Introduction

Tramadol hydrochloride (TMD), chemically (+)-trans-2-[(dimethyl-amino) methyl]-1-(3-methoxyphenyl) cyclohexanol, is a central analgesic agent for the treatment of severe moderate to chronic pain. Tramadol is also considered as an alternate to opiates for neuropathic pains. Tramadol also proves to produce antitussive, antidepressant, anti-inflammatory, and immune stimulatory effects [1, 2]. In humans, TMD metabolized by cytochrome P4502D6 to its phase 1 metabolites, namely, O-desmethyltramadol and N-desmethyltramadol. These are again metabolized to N,N-didesmethyltramadol, N,N,O-tridesmethyltramadol, and N, O-desmethyltramadol then further produce sulfate and glucuronic acid conjugates before excretion via kidneys in urine [3–5]. TMD is selective opiate agonist at *μ*-opioid receptors and inhibits reuptake of norepinephrine and serotonin [6]. TMD has plasma protein binding of about 20% and is rapidly absorbed with bioavailability of 65-70% after oral administration [7, 8]. As per the literature search, the analytical methods available for estimation of TMD with its desmethylates in plasma including liquid chromatography coupled to ultraviolet (UV) detector [9–11], fluorescence detector [12, 13], and tandem mass spectrometry (MS/MS) [14–22] are well reported.

Gabapentin (GBP), 1-(aminomethyl-1-cyclohexyl) acetic acid, is a structural analog of the inhibitory neurotransmitter amino butyric acid (GABA) which is a new generation effective antiepileptic drug for partial epileptic seizures with or without secondary generalization [23–25]. The GBP mechanism of action was not clearly defined, but described cellular actions are likely to be related to multiple concentration-dependent actions resulting in supremacy over seizure control [26]. It has been observed that GBP bioavailability varies greatly (inter- and intrasubjects) due to its active absorption by gut and renal excretion of unchanged drug. The bioavailability of a 600 mg oral dose was 49%; individual subjects may vary greatly from 5% to 74% [27, 28]. Gabapentin was proved to be beneficial in the treatment of neuropathic pain as well as postoperative pain following spinal surgery and hysterectomy [29]. Gabapentin in neuropathic pain models prevents mechanical and thermal allodynia and mechanical hyperalgesia. Though the mechanism of action of gabapentin in the treatment of neuropathic pain is not clear, it does not influence the same pathways as opioids or tricyclic depressants. Current evidence indicates that gabapentin affects voltage-gated calcium channels in the CNS [30, 31]. It was also reported that GBP was also effective in pain management because of neuralgia, diabetic neuropathy, multiple sclerosis, and neuropathic cancer pain in miscellaneous reports [32]. Several analytical methods were reported for the determination of GBP that includes high performance liquid chromatography (HPLC) coupled ultraviolet (UV) [33, 34], fluorescence detection [35], and mass spectrometry (MS) [36–42].

Fixed dose combination (FDC) of TMD with paracetamol for pain management in patients was available in market. The TMD combination with GBP is the present choice for doctors to treat pain carried by healthy nerves because of damaged tissues and damaged nerves (neuropathic). In present days, different combinations of TMD and GBP along with other analgesics like ibuprofen are under investigation [43]. The individual dosage forms for TMD and GBP were available around the globe but the FDC (TMD+ GBP) is commonly available in Latin America. The phase-IV clinical or bioequivalence studies are necessary for FDC approvals. Since no method was reported so far for simultaneous determination of TMD and GBP in human plasma, hence we aimed to develop specific and selective achiral assay for quantification of TMD and GBP in human plasma as per USFDA [44] and EMEA [45] bioanalytical method validation guidelines. The biological TMD metabolites (desmethlyates) measurement was not required to prove bioequivalence as per major health regulatory bodies; hence only parent drugs (TMD, GBP) are considered for method development. Finally, highly sensitive and repeatable method was developed for quantification of analytes in human plasma, useful to assess either efficacy or toxicity of both TMD and GBP (particularly) in various clinical situations. The present method is able to quantify the TMD and GBP at very low level (i.e., LLOQ 1 ng/mL and 10 ng/mL), which means that the established linear range is suitable to monitor TMD and GBP circulating levels across the relevant clinical range up to five terminal half-lives (t1/2), right from administration to approximate elimination (trough and subclinical concentrations) from the body [21, 22, 38].

## 2. Experimentation

### 2.1. Reference Standards and Reagents

The high purity reference standards of TMD, GBP, venlafaxine (VFX), and pregabalin (PGB) were procured from Clearsynth Labs Pvt. Ltd. (Mumbai, India). The HPLC grade methanol and acetonitrile are purchased from Thermo Fisher Scientific India Pvt. Ltd. (Mumbai, India). GR grade ammonium formate and ammonium acetate reagents were procured from Merck Specialities Pvt. Ltd. (Mumbai, India). Milli-Q water was collected from Milli-Q A10 gradient water purification system (Millipore, Bedford, MA, USA). Strata-X polymeric extraction cartridges (30 mg, 1cc) for solid phase extraction (SPE) are purchased from Phenomenex India Pvt. Ltd.

### 2.2. Analytical Instrumentation

An ultra flow prominence high performance liquid chromatography (UF-HPLC) coupled with tandem mass spectrometer (MS/MS-3200 model, Sciex, Canada) was used for analysis. The mass spectrometer was assembled with electro spray ionization (ESI) interface. The HPLC was supplied with LC-20AD binary pumps, 20A3 solvent degasser, column oven, and high-throughput SIL HTC auto sampler. After chromatographic separation, the positive polarity MS detection was performed in multiple reaction monitoring (MRM) mode. Analyst software 1.5.1 platform was used for data collection and hardware controlling.

### 2.3. Chromatographic Conditions

Analytical peak resolution was achieved on a Phenomenex, Kinetex PFP column (C18, 50 × 4.6 mm, 5 *μ*m) pumped with isocratic mobile phase consisting a mixture of 5 mM ammonium formate buffer (pH 3.0 ± 0.3), acetonitrile, and methanol in the ratio of 25: 50: 25 v/v. The flow rate was 0.8 mL/min. The auto sampler and column oven were programmed to maintain the set temperatures at 5°C and 35°C, respectively. Sample volume of 10 *μ*L was injected into the LC-MS/MS system. The total analytical run was 3.8 min.

### 2.4. MS/MS Compound and Source Dependent Conditions

The mass spectrometer was operated in positive mode to monitor parent→product ion (m/z) transitions of analytes (TMD, GBP) and their internal standards (ISTDs) (VFX, PGB). The specific details of MRM transitions and their respective mass spectrometer voltage values like declustering potential (DP), entrance potential (EP), collision energy (CE), and collision exit potential (CXP) used for quantification of respective analytes and ISTDs are summarized in Table 1. Manual tuning was performed to optimize the source dependent and compound dependent parameters to get highest credible intensities. The source dependent parameters like drying gas (GSI) and nebulizer gas (GS2) were set at 35 psi, 45 Psi duly. The turbo ion spray (TIS) temperature and ion spray voltage were set at 500°C and 4,500 V, respectively. The curtain gas (CUR) and collision associated dissociation gas (CAD) pressure were maintained at 30 psi and 8 psi. The unit resolution mode was employed in Q1 and Q3 (quadrupoles) with a dwell time of 300 milliseconds.

### 2.5. Standard Curve and Control Samples

Stock solutions of TMD and GBP were prepared in methanol and respective working (spiking) dilutions were made using diluent solution of methanol: water mixture (50:50,v/v). Separate stock weighing was done for preparation of calibration curve and quality control stock solutions. Calibration curves in range of 1-500 ng/mL and 10-6000 ng/mL were prepared for TMD and GBP, respectively. Quality control samples were made at concentration of 1 ng/mL lower limit of quality control (LLOQQC), 3 ng/mL lower quality control (LQC), 212 ng/mL middle quality control (MQC), 380 ng/mL high quality control (HQC), 1000 ng/mL diluted quality control (DQC) for TMD and 10 ng/mL (LLOQQC), 30 ng/mL (LQC), 2500 ng/mL (MQC), 4500 ng/mL (HQC), and 12000 ng/mL (DQC) for GBP. The 1% of respective working dilution was spiked into the total volume of plasma (for example, 10*μ*L of working solution was added to 990*μ*L of plasma, which is 1% to the total volume) to get above-mentioned concentrations for both the analytes. The long-term plasma stability samples at LQC and HQC level were prepared and stored at -70°C in ploy propylene tubes. The spiked samples were prepared freshly based on the validation experimentation plan. All the stock solutions and working dilutions were stored in refrigerator maintained at 2-8°C.

### 2.6. Bio Analytical Extraction Procedure

200 *μ*L of plasma sample was aliquoted using micropipette in to a 6mL polypropylene tube containing 100 *μ*L of ISTD solution (containing each 500 ng/mL of VFX and PGB) and then 0.2 mL of 100 mM ammonium acetate buffer as pretreatment solution was added. The resultant sample was briefly mixed and subjected to positive pressure solid phase extraction procedure using strata-X cartridges (30 mg/1 cc). The samples were loaded on cartridges which were already preconditioned with 1mL methanol and 1 mL Milli-Q water. Followed by loading, cartridge was washed with 1 mL 0.1% formic acid, 1 mL n-Hexane, and 1 mL methanol: water (5:95 v/v) solution step by step. Allow the cartridges to dry for about 3 min and then elute with 1 mL of 2% ammoniated methanol solution. The eluent solution was evaporated to dryness under gentle stream of nitrogen at a pressure of 20psi and at temperature of 50°C. The residue was reconstituted with 400 *μ*L of mobile phase and 10 *μ*L was injected into chromatographic system for analysis.

## 3. Method Validation

The developed method was validated to ensure method performance. The method was validated as per USFDA and EMEA guidelines. Method sensitivity, selectivity, linearity, precision, accuracy, recovery, matrix effect, dilution integrity, and analyte stability in biological matrix were evaluated. Each analytical run in validation begins with calibration curve and evenly distributed quality control samples at different levels based on standard experimental requirements.

### 3.1. System Suitability

Two injections of low standard solution and six injections of high standard solution containing both analytes (TMD, GBP) were injected to ensure system conditions. The low standard solution was injected to check the peak shape. The % CV for area ratio (analyte/ ISTD for both TMD, GBP) of high standard solution should be less than 4.

### 3.2. Biological Matrix Screening and Selectivity

The percentage of interference due to exogenous and endogenous components at retention times of analytes and ISTD was evaluated by processing eight different lots of blank plasma along with each two lots of hemolytic and lipemic plasma. The interference due to concomitant medication at retention time was also investigated by spiking paracetamol, ibuprofen, ranitidine, and ondansetron into drug free plasma at concentration equal to their available literature Cmax values. The interference observed at the retention times of analytes and ISTDs in blank plasma lots was compared against mean response of extracted LLOQ (n=6) samples. The observed interference should be less than 20% and 5% at analyte and ISTD retention times, respectively, when compared to mean response of extracted LLOQ samples.

### 3.3. Reproducibility (Precision) and Accuracy

At four different quality control levels (LLOQQC, LQC, MQC, and HQC, n=12) within day (intrabatch) and between day (interbatch, n=24) precision and accuracy of TMD, GBP was evaluated by calculating the %CV and %accuracy. In together six reproducibility batches were performed on two different days by two different analysts.

### 3.4. Effect of Matrix

The signal suppression or enhancement via ionization should be studied in mass spectrometric detection methods. To prove that, the method is free from matrix effect, postextraction response from 10 different lots (including each two lots of hemolytic and lipemic plasma) were compared with response of aqueous samples. The matrix effect was evaluated at LQC, HQC levels by calculating matrix factor of analyte and ISTD. Later ISTD normalized matrix factor was calculated by using matrix factor of analyte and ISTD. If ISTD normalized matrix factor value is 1, that indicates there is no suppression or enhancement due to the presence of matrix. If the value is less than 1, that indicates ion suppression or more than 1, that indicates ion enhancement. The acceptable limits for ISTD normalized matrix factor are 0.85-1.15.

### 3.5. Linearity of Analytes

The method linearity was assessed by constructing three eight-point calibration curves. A linear least-square regression analysis was applied for back calculated concentrations using weighing factors, none, 1/x, 1/x2. The weighing factor with least regression value is 1/x2; therefore 1/x2 was further used as weighing factor for constructing the calibration curves throughout the validation.

### 3.6. Extraction Recovery/Efficiency

Good extraction recovery was needed for accurate and reproducible results. Stable and consistent recovery was the basic requirement to achieve method sensitivity at limit of quantification (LOQ) level. The analyte recovery might be low or medium or 100% but it should be steady at all levels (LQC, MQC, HQC). Care should be taken while optimizing the procedure to achieve good extraction recovery. Relative recovery (RR) was evaluated at three different levels LQC, MQC, HQC (n=6) by comparing response in postspiked samples versus extracted samples. To evaluate true effect of matrix on recovery of analyte and ISTD (absolute recovery-AR), the response of extracted samples was also compared with aqueous samples. The recovery of analyte should not be more than 115%.

### 3.7. Stability of Analytes/ISTD

Stability of analytes (TMD, GBP) was evaluated in different experimental conditions based on the requirement of real time unknown sample analysis conditions like freeze and thaw stability (at −70°C), dry extract stability, spiked sample room temperature stability, auto sampler stability, long-term stability (at −70°C) and stability in whole human blood. For all the stability experiments six replicates of LQC, HQC samples were processed and analyzed against fresh calibration curve. The back calculated concentrations are compared to nominal concentration. Stability of aqueous samples was assessed by comparing the responses from high standard solutions prepared from stored aqueous stock solutions/working dilutions (at 2-8°C) with freshly prepared stock solutions/working dilutions.

## 4. Results and Discussion

### 4.1. Method Development

For efficient quantification and reliable results, it is prerequisite to give equal importance to optimize the chromatographic conditions, extraction procedure and mass spectrometric conditions. All analytes dissolved in methanol, individually infused into MS (mass spectrometer) source for tuning and then selected positive mode because of better intensity. The Q1 scan was performed to select the parent ion. The declustering potential (DP), entrance potential (EP) voltage values were further optimized to get highest intensity for parent ion. After that, collision energy (CE), collision cell exit potential (CXP) values were optimized in MSMS scan to select product ion for TMD, GBP, PGB and VFX. The observed [M+H]^+^ peaks (parent ion) and respective consistent product ions were selected for mass spectrometric transitions (Q1/Q3) in MRM (multiple reaction monitoring) mode for quantification. The selected transitions and optimized voltage values were shown in Table 1. The unit resolution mode with a dwell time of 300 milliseconds was used for each MRM transition channel.

**Figure 1:**
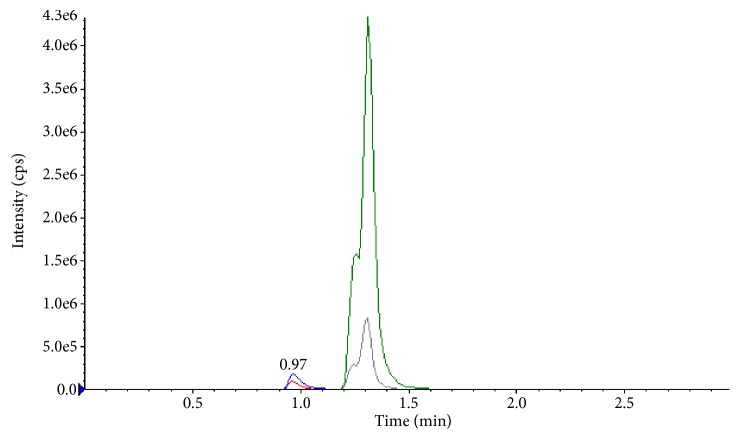
Chromatogram of analytes/ISTDs (LLOQ level) injected normal gemini C_18_ column (50 × 4.6 mm, 5 *μ*m).

**Figure 2:**
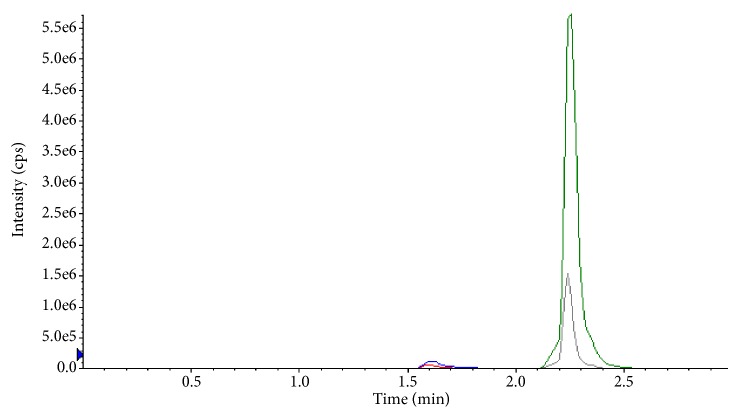
Chromatogram of analytes/ISTDs injected on high purity C_18_ column (100 × 4.6 mm, 3.5 *μ*m).

Several analytical bonded stationary phases of C_18_ and C_8_ were checked and retention times of analytes are overlapped. Initially, aqueous solution of LLOQ level was injected into normal gemini C_18_ (50 × 4.6 mm, 5 *μ*m) column, but the observed peak resolution was not good and peak intensity is very low, the identical chromatogram of LLOQ solution in gemini column was shown in Figure 1. Then sample solution was injected into thermo high purity C_18_ (100 × 4.6 mm, 3.5 *μ*m), column to improve the peak shape. The observed peak resolution was comparatively good with low intensity. The representative chromatogram was shown in Figure 2. The better peak shape and resolution with required sensitivity was achieved on Phenomenex, PFP (50 × 4.6 mm, 2.6 *μ*m) column may be because of its combining C_18_ retention properties and unique aromatic PFP selectivity. A medium strength buffer 5 mM ammonium formate gives high signal to noise ratio with negligible baseline noise at LLOQ level.

**Figure 3:**
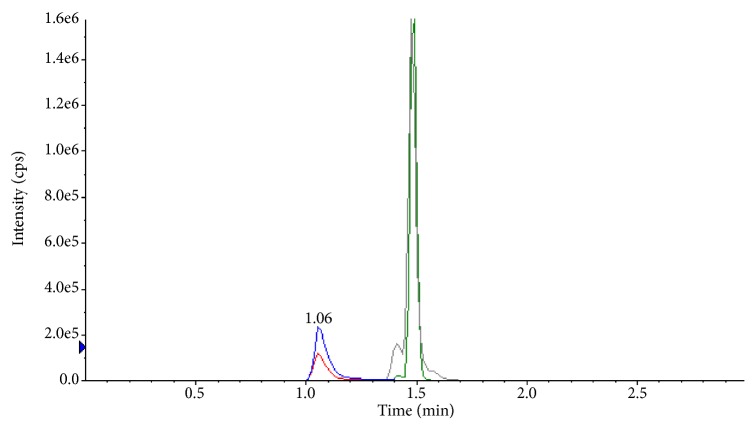
Chromatogram of analytes/ISTDs with high base line noise (liquid-liquid extraction).

In sample extraction, liquid-liquid and solid phase extraction techniques were investigated. In liquid-liquid extraction high base line was observed because of possible matrix contaminants. The representative chromatogram is shown in Figure 3. Finally, solid phase extraction was selected due to its high consistent extraction recoveries with no matrix effect and cleaner extracts. Phenomenex Strata-X cartridges with 5% methanol wash produced side peaks in chromatography and low recovery was observed with GBP. An acidic wash with 0.1 % formic acid increases the GBP recovery, followed by n-hexane wash to eliminate nonpolar interferences prior to the elution resolved the side peaks issue. Method was strictly optimized to get similar recoveries for analytes and ISTDs. The nearly same % recovery results for analytes and ISTDs with acceptable ISTD normalized factor values of the method assure reproducible quantification.

### 4.2. Selectivity

Eight plasma lots along with each two different lots of hemolytic and lipemic plasma were processed and injected for LC-MS/MS analysis. Similar chromatography was observed with no significant interference at the retention times of analytes and ISTDs in all analyzed blank lots, which indicates that the developed method was highly selective.

### 4.3. Linearity

Three calibration curves were generated by plotting the area ratios (analyte response/ ISTD response) on y-axis and concentration on x-axis. The plot was linear throughout the established calibration ranges, 1-500 ng/mL for TMD and 10-6000 ng/mL for GBP. The slope values are consistent and regression values were found to be more than 0.99. The back calculated concentrations for individual calibration standards are meeting acceptance criteria for accuracy (±15 %) and precision (≤ 15 %).

### 4.4. Sensitivity

Six replicates of LLOQ samples were processed and analyzed against calibration curve. The accuracy and precision values were 91.3 % and 3.8 % for TMD and 98.6 % and 2.9 % for GBP. The observed signal to noise ratio is more than 5:1 for both the analytes.

### 4.5. Precision and Accuracy

**Figure 4:**
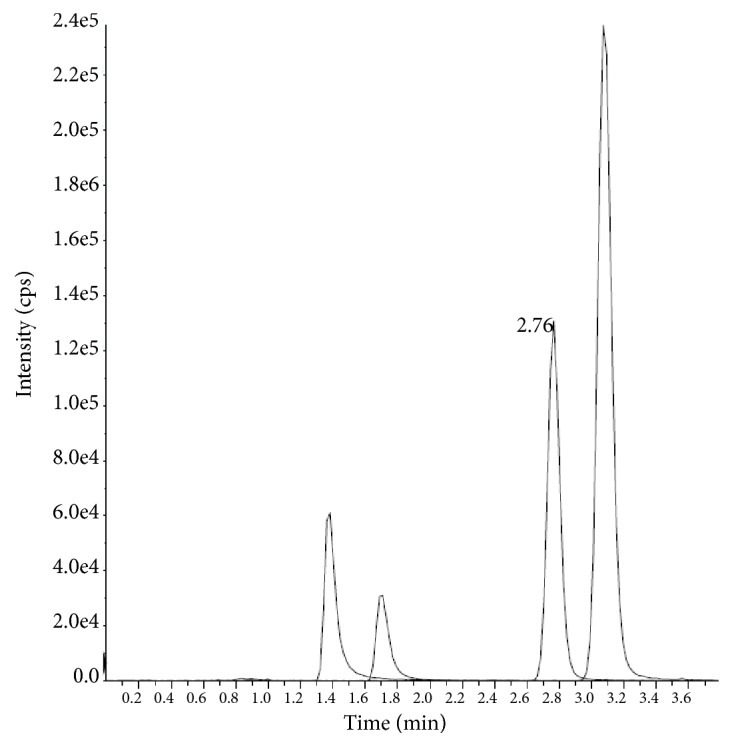
Chromatogram of analytes/ISTDs at LLOQQC level.

Accuracy and reproducibility results of intra- and interbatches of TMD and GBP were reported in Tables 2 and 3, respectively. The intra- and interbatch accuracy values were in the range of 92.3%-99.2% and intra and interbatch precision were found to be less than 10.9 % and 6.7 % for TMD and GBP. The chromatogram at LLOQ level was shown in Figure 4.

### 4.6. Effect of Matrix

In general consideration, effect of matrix does not influence peak resolution due to MS selectivity. However, in this method sufficient resolution between the analytes (TMD, GBP) was established chromatographically. Matrix effect was evaluated in 10 different lots. The obtained ISTD normalized matrix factor values for both the analytes were in the range of 0.93 to 1.06. The precision values for ISTD normalized factor at LQC and HQC level were 2.0% and 3.6% for TMD and 2.3% and 4.0 % for GBP. The results are presented in Table 4.

### 4.7. Recovery

Absolute and relative recovery of analytes and ISTDs was evaluated. The mean recovery results of TMD, GBP, VFX, and PGB are represented in Table 5.

### 4.8. Dilution Integrity

Precision and accuracy of diluted plasma samples were assessed at 1:4 dilution. The DQC (dilution quality control) was prepared by spiking at a concentration equal to two times of high level calibration standard of proposed range for TMD and GBP, respectively. Then 1/4^th^ volume of plasma aliquot was diluted with drug free plasma and analyzed against calibration curve. The accuracy values were in the range of 92.4%-106.5 % and %CV was less than 3.2% for TMD and GBP.

### 4.9. Stability

All the stock solutions and stock dilutions were stable for 21 days at refrigerated storage maintained at 2-8°C. The processed stability samples in plasma at LQC and HQC levels were analyzed against freshly prepared calibration curve. The stability data results are given in Table 6. TMD and GBP were stable in plasma at room temperature for about 16 h and for 5 freeze and thaw cycles. The established stability time for TMD and GBP was 41 h and 52 h for auto sampler and dry extract stabilities. The analytes were found to be stable for 2.5 h in blood. The long-term stability was evaluated and analytes were stable for 32 days at −70°C.

## 5. Conclusion

Full method validation was carried out using screened and pooled human plasma to ensure that developed procedure is accurate and precise for estimation of TMD and GBP simultaneously. The high-throughput LC-ESI-MS/MS method is sensitive and specific. The recovery, precision, and accuracy results were reproducible over the proposed calibration ranges for TMD and GBP. The shorter runtime allows the analysis of more samples (~300) per day. The method can be readily used by scientific community for the application of sample analysis for therapeutic monitoring/pharmacokinetic or bioequivalence studies.

## Figures and Tables

**Table 1:** MRM and mass spectrometer voltage details (TMD, GBP) and IS (VFX, PGB).

Name of the molecule	MRM Transition (Q1/Q3)	DP	EP	CE	CXP
TMD	264.2/58.1	50	10	22	15

GBP	172.2/154.2	38	10	27	10

VFX	278.3/121.1	70	10	27	8

PGB	160.2/97.1	90	10	23	11

**Table 2:** Precision and accuracy results of TMD.

QC name/nominal concentration	TMD			
	Intra batch (n=12)		Inter batch (n=24)	
	% Accuracy	% CV	% Accuracy	% CV
LLOQQC/ 1 ng/mL	92.3	10.9	91.8	9.8

LQC/ 3 ng/mL	97.3	8.6	95.2	6.1

MQC/ 212 ng/mL	94.9	3.8	96.7	5.5

HQC/ 380 ng/mL	99.2	5.8	97.6	4.2

**Table 3:** Precision and accuracy results of GBP.

QC name/nominal concentration	GBP			
	Intra batch (n=12)		Inter batch (n=24)	
	% Accuracy	% CV	% Accuracy	% CV
LLOQQC/ 10 ng/mL	96.7	6.7	98.4	3.8

LQC/ 30 ng/ mL	93.5	5.8	97.9	6.6

MQC/ 2500 ng/mL	99.1	3.2	94.1	2.9

HQC/ 4500 ng/mL	95.6	2.9	100.9	5.3

**Table 4:** Matrix effect results of TMD and GBP.

Blank plasma lots	TMD (ISNMF)		GBP (ISNMF)	
	LQC	HQC	LQC	HQC
LOT-1	0.99	0.96	0.93	1.02

LOT-2	0.98	0.97	0.94	1.05

LOT-3	0.98	0.96	0.92	0.94

LOT-4	0.97	0.95	0.96	0.98

LOT-5	1.02	0.99	0.99	0.96

LOT-6	1.01	1.06	0.97	0.97

LOT-7 hemolytic	0.99	1.01	0.98	0.99

LOT-8 hemolytic	1.03	0.99	0.96	1.04

LOT-9 lipemic	0.98	0.94	0.96	0.94

LOT-10 lipemic	0.99	0.97	0.94	0.96

Mean	0.994	0.980	0.955	0.985

SD	0.0196	0.0350	0.0222	0.0395

%CV	2.0	3.6	2.3	4.0

ISTDNMF: internal standard normalised matrix factor.

**Table 5:** Recovery results of TMD and GBP.

Sample name	% Mean recovery absolute		% Mean recovery relative	
	TMD	GBP	TMD	GBP
LQC	81.5	83.5	83.8	86.4

MQC	78.4	80.5	81.2	82.1

HQC	84.7	79.9	78.9	82.2

VFX at MQC level	85.4		79.1	

PGB at MQC level	81.9		82.4	

**Table 6:** Stability results of TMD and GBP.

Stability experiment	Stability condition	%Mean stability			
		TDL		FNS	
		LQC	HQC	LQC	HQC
Auto sampler stability	41 h at 5°C	98.1	96.4	96.1	106.7

Free and thaw stability	5 cycles at -70°C ± 15°C	97.1	100.1	99.9	96.1

Dry extract stability	52 h at 2-8°C	94.8	96.8	101.2	96.6

Room temperature stability	16 h at room temperature at 25°C ± 5°C	94.1	102.8	91.7	97.2

Long term stability	32 days at -70°C ± 15°C	99.6	95.7	98.8	95.5

Stability in blood	2.5 h room temperature at 25°C ± 5°C	96.4	99.1	103.2	97.6

## Data Availability

The data used to support the findings of this study are available from the corresponding author upon request.
